# The Local Government Board on Country Water Supplies

**Published:** 1898-01-15

**Authors:** 


THE LOCAL GOVERNMENT BOARD ON COUNTRY WATER SUPPLIES.

The Local Government Board have issued an order calling the attention of town, urban, district, and rural district councils to the subject of their water supplies, and it is satisfactory to note that it is therein definitely laid down that where the council have themselves constructed or purchased any water works it is their duty, in pursuance of section 55 of the Public Health Act, 1875, to provide a supply of "pure and wholesome" water, and in order to fulfil this obligation it behoves them to exercise every precaution to secure that the water which they deliver to the consumers shall be protected from risk of contamination. The order goes on further to state that the council are "under a responsibility for the wholesomeness of the water which they themselves supply." Is it not, then, an anomaly that water companies which, although private concerns, are established by Act of Parliament, should be allowed to act as if they were free from such responsibility?

